# Association between tumor necrosis factor alpha and obstructive sleep apnea in adults: a meta-analysis update

**DOI:** 10.1186/s12890-020-01253-0

**Published:** 2020-08-12

**Authors:** Yuan Cao, Yali Song, Pu Ning, Liyu Zhang, Shuang Wu, Juan Quan, Qiao Li

**Affiliations:** 1 Department of Pulmonary and Critical Care Medicine, The Second Affiliated Hospital of Xi’an Jiaotong University (Xibei Hospital), Xi’an, Shaanxi China; 2 Department of Laboratory Medicine, West China Hospital, Sichuan University, Chengdu, Sichuan China; 3 Institute of Pediatric Diseases, The Affiliated Children Hospital of Xi’an Jiaotong University, Xi’an, Shaanxi China; 4 Clinical Laboratory, The Affiliated Children Hospital of Xi’an Jiaotong University, Xi’an, Shaanxi China; 5 Department of Ultrasound, School of Medicine, The Second Affiliated Hospital of Zhejiang University, Zhejiang, Hangzhou China

**Keywords:** Obstructive sleep apnea, Meta-analysis, Tumor necrosis factor-α

## Abstract

**Background:**

Tumor necrosis factor-α (TNF-α) has been reported to play a part in the development of obstructive sleep apnea (OSA) and its complications. However, the relationship between TNF-α and OSA still remains inconclusive. We aimed to systematically review and synthesize studies published to date on association between the two in adults.

**Methods:**

We searched for English-language articles containing original human data from case-control study studies in adults≥18 years of age. The selection criteria were set according to the PICOS framework. Articles were independently reviewed by three investigators. Data regarding demographics, clinical characteristics, and TNF-α levels were obtained. A random-effects model was applied to evaluate the overall effect sizes by calculating standard mean difference (SMD) and its 95% confidence intervals (*CI*s).

**Results:**

Of 393 identified abstracts, 50 articles (3503 OSA patients and 3379 health controls) were ultimately included in this meta-analysis. The results indicated that the TNF-α level in patients with OSA was 1.77 (95%CI, 1.37 to 2.17, *I*^*2*^ = 97.8%, *P* < 0.0001) times higher than in the control group. Subgroup analyses showed a positive correlation between the level of TNF-α and OSA severity. According to meta-regression, we noted that aging significantly predicted an increased effect size of TNF-α level in OSA patients (*P* < 0.007).

**Conclusion:**

This study identified a significant association between OSA and elevated TNF-α level in adults. Meanwhile, TNF-α levels were consistently correlated with severity of OSA, which indicated it might be a promising biomarker for the development of OSA. However, well-designed, large-scale, case-control cohorts are needed to better understand the relationship of TNF-α in the context of adult OSA.

## Background

Obstructive sleep apnea (OSA) is a common chronic respiratory disease, and presented a high prevalence in the general population. It estimated that the overall prevalence of symptomatic OSA is 15 to 50% in general population, and the rates are still increasing [1]. OSA is characterized by repetitive collapse of the upper airway during sleep, which leads to intermittent hypoxia and arousals during sleep, moreover, it increases the risks of complications of cardiovascular, neurocognitive disturbance, and metabolic morbidities [2, 3]. Furthermore, long-term hypoxia will increase the systemic inflammation of patients with OSA. With the accumulation of these increasing inflammatory cytokines and mediators, it will further contribute to the onset of OSA.

Tumor Necrosis Factor Alpha (TNF-α) is a pro-inflammatory cytokine which usually is secreted by mononuclear-macrophage, natural killer cells, and other immune cells [4, 5]. It plays a crucial role in host defense and mediates the pathogenesis of a wide spectrum of disease processes such as cancers, infectious diseases, autoimmune diseases, cardiovascular diseases and atherosclerosis [5]. TNF-α has been implicated in the regulation of sleep by activating NF-κB pathways, and leading to upregulate of adenosine A1 receptor, cyclooxygenase-2, and NO synthase, which thought to be involved in sleep regulation [6, 7]. And, TNF-α antagonist has proven to attenuate cognitive and behavioral disturbances, thus, ameliorate the progression of OSA [8, 9]. Previous literatures had utilized the levels of TNF-α in the blood or excretion of OSA patients as an evaluation indicator of occurrence and disease development, and usually increased compared with healthy subjects or decreased after treatment [10, 11]. It was also mentioned that TNF-α could assess the degree of OSA. However, due to the range of nations, regions, severity in OSA patients, sample size, comorbidity of patients, specimens, and methods of detection varied among different studies, with the majority of studies lacking a uniform procedure, thus had less adequate statistical power to clarify the relationship between TNF-α and OSA.

To comprehensively understand TNF-α in OSA, we performed this meta-analysis in patients with OSA to evaluate whether the levels of TNF-α were higher than in healthy controls and, to identify the association between TNF-α and OSA in adults.

## Methods

### Literature search

We systematically searched four electronic databases (PubMed, Web of Science, EMBASE, and Cochrane library database CENTRAL) through September 2019. The search terms included [‘Respiration Disorders, Sleep Apnea Syndromes’ or ‘obstructive Sleep Apnea’ or ‘chronic obstructive airway disease’ or ‘OSA’ or ‘OSAHS’] and [‘Tumor necrosis factor-a’ or ‘Tumor necrosis factor alpha’ or ‘TNF-α’] and (‘systemic inflammation’ or ‘biological markers’). Only articles published in English were included. We also went through the references of eligible studies and manually review articles to identify possible relevant publications.

### Study selection

The PICOS (population, intervention, comparison, outcomes, and study design) framework was introduced as inclusion criteria. All articles had to meet certain criteria (see supplementary Table 1). OSA patients were diagnosed according to the clinical practice guideline from the American College of Physicians [12]. Studies eligible for the meta-analysis included those that measured the levels TNF-α in OSA or controls and in which the degrees of TNF-α should determine using pg/ml or calculate to accordant unit.

The exclusion criteria include studies that included participants with a history or diagnosis of other respiratory, cardiovascular and endocrine diseases overlapping with OSA; pediatric or adolescent studies, patients who received nutritional support or undergoing therapy (medication, operation, continuous positive airway pressure); case reports or those articles lacking statistical data; articles without control group (articles with only case groups could be included in subgroup analysis).

### Data extraction

Two authors (Yuan Cao and Qiao Li) independently screened the literature and extracted data to ensure that the screening core criteria and data gathering were consistent. If the opinions were different, further discussion was carried out or an additional person (Yali Song) was invited to participate in discussions until a consensus was reached. By designing a data extraction form and following items were drawn from the qualified articles: the first author, year of publication, nationality, sample size, gender, BMI, study design, levels of TNF-α.

The quality of the included studies was evaluated according to the Newcastle-Ottawa Scale (NOS). Two authors independently performed the analyses, and consensus was reached on all decisions.

### Statistical analysis and data synthesis

All data were entered into STATA, version 13.0 for meta-analysis. Data were presented as mean ± SD to evaluate the relationships between TNF-α and OSA. If the data were presented as mean (95% confidence interval), median (range), or median (interquartile), the formula from the published articles was used to convert the data and calculate SD [13, 14]. The heterogeneity of the studies was measured using the *I*^*2*^ statistic, with the level of significance set at *P* < 0.05, and *I*^*2*^ values of 25, 50, and 75% representing low, moderate, and high heterogeneity, respectively. Considering the probable heterogeneity of the studies, a random-effects model was performed in the meta-analysis following the approach of DerSimonian and Laird [15]. Likewise, a subgroup analysis was conducted to compare observed effects between OSA and controls, with *P* < 0.05 denoting statistical significance. Publication bias was assessed by funnel plot and using Egger’s test, and then conducted a trim and fill analysis was conducted, as well as a sensitivity analysis to adjust asymmetry of funnel plot to address the problem of publication bias.

## Results

### Characteristics of the individual studies

**Fig. 1:**
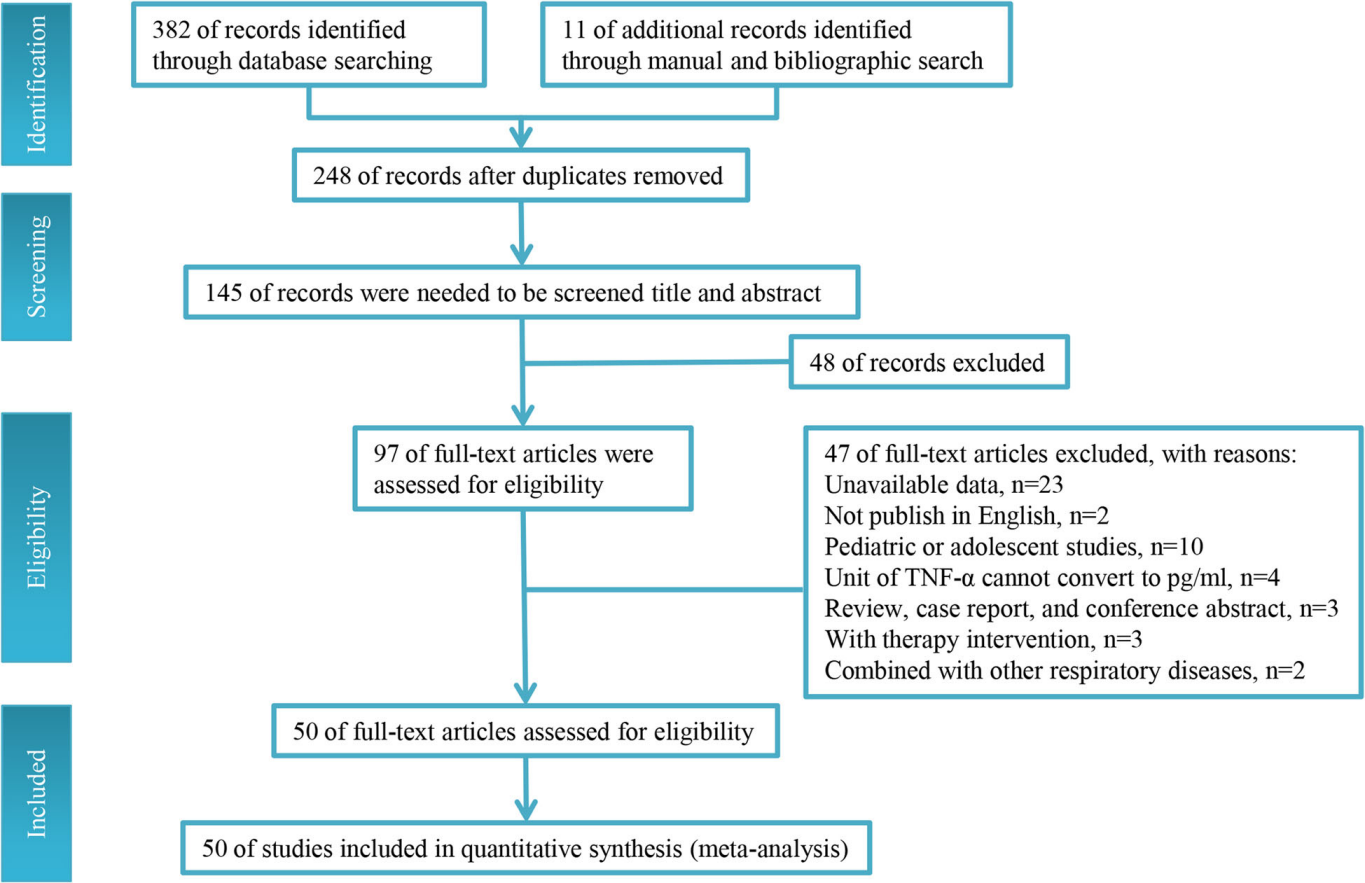
Flow diagram of the literature search process

Our database search initially revealed a total of 248 articles. After checked for duplication, 248 studies were included in our meta-analysis, in which, 145 articles need further screen. After reading the title and abstract of each article, 48 articles were excluded. The remaining 97 articles were screened by the inclusion and exclusion criteria. Finally, 50 articles reporting data from 3503 OSA patients and 3379 health controls were included in the meta-analysis (Fig. 1).

The studies were published from 1993 through March 2019. Of those, 25 studies were conducted in Asia, 18 in Europe, and 7 in America. Among all the studies, 14 concentrated on the severity of OSA, mild OSA was reported in 7 studies, mild to moderate was reported in 6 studies, moderate OSA was reported in 5 studies, moderate to severe OSA was reported in 13 studies, and severe OSA was reported in 5 studies.

The baseline characteristics and NOS scores of each study included in our meta-analysis are listed in Table 1 [10, 11, 16–64]. Forty-four studies scored more than 5 stars in NOS which suggested a high-quality study. The sample size, mean age, gender, mean BMI, and levels of TNF-α of individuals in the studies are provided in Table 1.

**Table 1:** Characteristics of studies included in the meta-analysis Study

Study	Year	Country	Sample size	Sample		mean age		Gender (male/female)		BMI (kg/m^2^)		TNF (pg/ml)		NOS
				Case	Control	Case	Control	Case	Control	Case	Control	Case	Control	
Ming	2019	China	876	684	192	51.34 ± 5.16	52.18 ± 4.51	446/238	128/64	24.1 to 30.5	NR	31.2 ± 5.3	12.1 ± 1.1	9
Sundbom	2018	Sweden	333	109	224	55.6 ± 8.8	47.7 ± 11.3	0/109	0/224	28.2 ± 4.8	25.4 ± 4.1	Mean (95%CI) 2.1 (1.8, 2.4)	Mean (95%CI) 2.0 (1.8, 2.2)	7
Bhatt	2018	India	240	47 (without **NAFLD**)	25 (without **NAFLD**)	44.2 ± 9.1	41 ± 8.5	25/22	17/8	32.5 ± 6.9	28.5 ± 8.6	Log (3.6 ± 0.14)	Log(2.86 ± 0.2)	8
				124 (with **NAFLD**)	44 (with **NAFLD**)	44.8 ± 9.1	39.5 ± 10.5	64/60	26/18	33.3 ± 7.9	31.0 ± 8.3	Log (3.86 ± 0.18)	Log(3.2 ± 0.06)	
Kong	2018	China	90	50 (Mild 10, Moderate 15, Severe 25)	40	54.34 ± 14.38 (Mild 59.60 ± 15.13, Moderate 56.73 ± 15.93, Severe 52.27 ± 13.54)	50.42 ± 8.35	34/16	31/9	26.86 ± 3.12 (Mild 27.04 ± 2.58, Moderate 26.81 ± 3.80, Severe 27.28 ± 3.16)	22.26 ± 3.54	327.34 ± 46.81 (Mild 274.64 ± 4.91, Moderate 308.75 ± 42.48, Severe 341.28 ± 44.80)	307.95 ± 27.15	7
Bozic	2018	Croatia	75	50 (Moderate 25, Severe 25)	25	Moderate 53.92 ± 10.75, Severe 52.04 ± 13.11	52.52 ± 10.18	50/0	25/0	Moderate 28.42 ± 2.57, Severe 29.30 ± 2.74	27.78 ± 2.23	Moderate 5.79 ± 1.44, Severe 8.67 ± 2.41	2.35 ± 1.25	7
Ugur	2018	Turkey	108	63	45	median (range) 46 (20–81)	median (range) 45 (20–70)	NR	NR	median (range) 25 (17–49)	median (range) 24.8 (15–33)	median (range) 2710 (80–59,520)	median (range) 660 (80–23,140)	8
Heizati	2017	China	159	28	54	44.00 ± 8.26	44.94 ± 8.33	28/0	54/0	26.09 ± 1.75	25.30 ± 1.79	median (interquartile) 0.302 (0.177–0.436)	median (interquartile) 0.298 (0.182–0.481)	8
				40 (obese)	37 (obese)	44.40 ± 8.47	47.35 ± 6.75	40/0	37/0	30.69 ± 2.03	30.30 ± 1.97	median (interquartile) 0.281 (0.149–0.470)	median (interquartile) 0.281 (0.185–0.426)	
Jin	2017	China	150	100	50	55.284 ± 7.128	56.131 ± 6.210	82/18	37/13	26.746 ± 3.500	25.196 ± 2.449	37.67 ± 0.21	29.15 ± 1.74	6
Hirotsu	2017	Brazil	1042	193	275	47.0 ± 1.0	36.2 ± 0.8	193/0	275/0	29.0 ± 0.4	24.9 ± 0.2	10.91 ± 0.44	10.84 ± 0.38	5
				149	425	55.7 ± 1	39.5 ± 0.6	0/149	0/425	30.4 ± 0.5	25.8 ± 0.2	10.95 ± 0.52	9.2 ± 0.29	
Tirado*	2017	Spain	66	Mild 20, Moderate 16, severe 30	NO	Mild 35.8 ± 8.43 Moderate 45.6 ± 9.08 Severe 44.5 ± 11.0	NO	Mild 2/18, Moderate 2/14, Severe 8/22	NO	Mild 46.3 ± 7.05, Moderate 43.1 ± 4.43, Severe 46.4 ± 6.18	NO	Mild 2.33 ± 0.72, Moderate 3.57 ± 2.55, Severe 2.33 ± 1.04	NO	5
Gamsiz-Isik	2016	Turkey	163	83 (Mild 16, Moderate to severe 67)	80	46.87 ± 8.21	44.23 ± 9.83	65/18	57/23	30.91 ± 3.31	31.53 ± 3.44	11.5 ± 3.11 (Mild 11.5 ± 4.60, Moderate to severe 10.3 ± 2.59)	11.25 ± 4.0	6
Vicente	2016	Spain	115	89 (Mild to Moderate 42 Severe 47)	26	median (interquartile) 44(36–56)	median (interquartile) 44(38–53)	62/27	16/10	median (interquartile)29.1 (27.1, 33.9)	median (interquartile) 27.9 (25.2, 31.1)	median (interquartile) Mild to Moderate: Plasma 4.54 (4.09–5.25), Lavage 1.8 (1.1–3.2); Severe: Plasma 4.46 (4.21–5.06), Lavage 2.1 (1.7–4.2)	median (interquartile) Plasma 4.35 (4.01–4.99); Lavage 1.2 (0.8–2.1)	5
Ifergane	2016	Israel	43	21	22	66.0 ± 9.9	66.1 ± 13.1	8/13	5/17	29.6 ± 4.3	26.8 ± 4.3	6.39 ± 5.00	3.57 ± 1.87	5
Nizam	2015	Turkey	52	39 (Mild to Moderate 17, Severe 22)	13	Mild to Moderate 49.88 ± 11.47, Severe 45.36 ± 9.81	43.23 ± 9.08	Mild to Moderate 9/8, Severe 18/4	5/8	Mild to Moderate 31.85 ± 5.32 Severe34.18 ± 7.24	31.71 ± 4.56	Mild to Moderate: Serum 86.7 ± 40.5, Saliva 10.6 ± 10.1; Severe Serum 99.5 ± 64.63, Saliva 10.2 ± 9.8	Serum 91.3 ± 64.6; Saliva 6.8 ± 2.2	9
Leon-Cabrera	2015	Mexico	39	29	10	37.2 ± 11.4	43.4 ± 11.5	4/25	8/2	45.2 ± 8.4	23.6 ± 2.1	337.9 ± 67.8	270.2 ± 31.7	9
Jiang	2015	China	229	135	94	48.7 ± 12.1	47.2 ± 13.5	80/55	55/39	27.48 ± 2.56	27.52 ± 2.58	765.77 ± 64.04(Mild 545.36 ± 54.06, Moderate 764.48 ± 63.28; Severe 836.72 ± 71.06)	232.24 ± 31.5	6
Thunström	2015	Sweden	329	Moderate to Severe 234	95	65.3 ± 7.1	61.4 ± 9.5	204/30	71/24	26.8 ± 2.1	25.2 ± 2.5	median (interquartile) 5.0 (3.4–7.0)	median (interquartile) 4.2 (3.0–6.0)	8
De Santis	2015	Italy	50	26	24	41.8 ± 7.4	43.7 ± 8.2	17/9	16/8	33.0 ± 5.2	30.8 ± 4.3	122.2 ± 12.0	80.2 ± 18.3	4
Salord	2014	Spain	39	26	13	median (interquartile) 45(39–51)	median (interquartile) 39(31–46)	6/20	4/9	median (interquartile) 45.1(42–45)	median (interquartile) 44.4(41–47)	median (interquartile) 9.8 (8.0, 12.3)	median (interquartile) 9.1 (7.5, 11.5)	5
Ciccone	2014	Italy	120	80 (Mild26,moderteto Severe 54)	40	Mild 53.65 ± 11.47, Moderate to Severe 52.33 ± 10.19	52.27 ± 10.52	Mild 23/3, Moderate to Severe 45/9	34/6	Mild 28.13 ± 2.7, Moderate to Severe 28.8 ± 3.03	28.24 ± 2.7	Mild 14.42 ± 3.29, Moderate to Severe 22.83 ± 3.85	12.53 ± 3.48	5
Yadav	2014	UK	41	20	21	49 ± 10	45 ± 9	3/17	5/16	52 ± 6	50 ± 8	median (interquartile) 87.2 (12.4, 133.8)	median (interquartile) 15.5 (7.2, 38.2)	6
Chen	2013	China	64	44 (Mild23; Moderate 21)	20	Mild 40 ± 11, Moderate 45 ± 13	42 ± 11	Mild 17/6, Moderate 16/5	15/5	Mild 27.5 ± 4.2, Moderate 26.7 ± 2.8	26 ± 3.3	median (interquartile) Mild 2.8 (0.8, 4.1), Moderate 3.8 (2.5, 9.9)	median (interquartile) 1.2 (0.5,1.7)	7
Doufas	2013	USA	48	33	15	median (range) 34(19–54)	median (range) 31(19–52)	33/0	15/0	median (range) 26(20–33)	median (range) 24(20–32)	median (range) 7.88 (5.39, 31.69)	median (range) 7.77 (4.57,14.57)	5
Hargens	2013	USA	30	12 (obese)	18 (obese)	22.8 ± 0.8	22.5 ± 0.7	12/0	18/0	32.4 ± 1.0	31.6 ± 1.1	0.95 ± 0.07	0.86 ± 0.05	6
Yang	2013	China	50	25	25	54 ± 7	53 ± 7	23/2	23/2	27.39 ± 2.91	26.27 ± 1.9	12.55 ± 8.09	5.12 ± 1.23	7
Fornadi	2012	Germany	100	25	75	54 ± 12	50 ± 13	20/5	60/15	29 ± 5	26 ± 5	median (interquartile) 2.2 (1.5–2.8)	median (interquartile) 1.9 (1.3–2.6)	6
Medeiros	2012	Brazil	65	50 (Mild to Moderate 15; Severe 35)	15	Mild to Moderate 62.62 ± 9, Severe 65 ± 7.2	62.5 ± 8.4	Mild to Moderate 11/4;Severe 20/15	6/9	Mild to Moderate 24.5 ± 3.8, Severe25.9 ± 4.1	25.81 ± 4.04	Mild to Moderate 0.84 ± 1.8, Severe 2.09 ± 7.3	0.32 ± 0.77	6
Qian	2012	China	110	70 (40 with hypertension; 30 without)	40	With hyperten 46.9 ± 7.0, without 45.0 ± 9.0	46.3 ± 8.1	70/0	40/0	With hyperten 28.2 ± 2.5, without 29.4 ± 2.1	24.1 ± 2.3	With hyperten 1290 ± 220, without 1150 ± 370	1140 ± 400	6
Kim	2010	Korea	59	37 (Moderate 9, Severe 28)	22	Moderate 38 ± 15.04, Severe 42 ± 10.77	26 ± 6.91	37/0	22/0	Moderate 24.43 ± 2.45, Severe 28.69 ± 4.05	23.88 ± 2.30	Moderate 14.56 ± 5.61, Severe 15.32 ± 6.8	14.4 ± 4.13	5
Li	2010	China	417	Normotensive 113; hypertension 134	97; 73	45.45 ± 8.63; 46.10 ± 9.41	44.16 ± 8.59; 45.97 ± 9.06	85/28; 101/33	74/23; 54/19	27.84 ± 3.44;28.91 ± 3.24	26.85 ± 3.78;27.7 ± 3.01	19,980 ± 8480; 22,850 ± 8980	13,100 ± 4280; 17,320 ± 7020	8
Steiropoulos	2010	Greece	61	38	23	45.5 ± 10.5	43.7 ± 6.7	33/5	17/6	36.4 ± 7.4	34.5 ± 3.7	6.72 ± 3.72	3.94 ± 1.34	8
Tamaki	2009	Japan	46	33 (Mild to Moderate 13, Severe 20)	13	Mild to Moderate 56.1 ± 8.7, Severe 50.5 ± 12.2	35.5 ± 9.7	Mild to Moderate 11/2, Severe 19/1	12/1	Mild to Moderate 24.6 ± 2.7, Severe 30.7 ± 5.8	23.6 ± 2.6	Mild to Moderate 22,700 ± 4100, Severe 30,200 ± 6900	17,300 ± 4400	6
Sahlman	2009	Finland	124	84	40	50.4 ± 9.3	45.6 ± 11.5	64/20	25/15	32.5 ± 3.3	31.5 ± 3.5	1.54 ± 1.75	1.17 ± 1.58	8
Bhushan	2009	India	207	104	103	46.18 ± 10.7	44 ± 10	84/20	65/38	31.48 ± 4.26	30.94 ± 4.27	Log (3.6 ± 0.8)	Log (3.3 ± 0.6)	7
Carneiro	2009	Brazil	29	16	13	40.1 ± 2.8	38.8 ± 3.3	16/0	13/0	46.9 ± 2.0	42.8 ± 1.3	10.7 ± 0.44	7.5 ± 0.44	7
Thomopoulos	2009	Greece	132	62 (hypertension)	70 (hypertension)	48.1 ± 7.6	48.1 ± 3.9	49//13	56/14	31.9 ± 4.9	32.1 ± 3.0	Log(0.33 ± 0.27)	Log(0.10 ± 0.23)	5
Li	2009	China	90	68 (Mild 22, Moderate 22, Severe 24)	22	Mild 48 ± 12, Moderate 44 ± 13, Severe 44 ± 8	43 ± 9.3	Mild 15/7, Moderate 18/4, Severe 17/7	14/8	Mild 25.7 ± 4.2, Moderate 28.8 ± 5.3, Severe 28.67 ± 4.2	23.3 ± 2.0	Serum: Mild 102.3 ± 11.3, Moderate 125 ± 11.9, Severe 132.1 ± 10.8 EBC: Mild 96.1 ± 8.2, Moderate 116.7 ± 11.1, Severe 128.2 ± 8.8	Serum: 87.3 ± 6.1; EBC: 83.7 ± 4.1	9
Antonopoulou	2008	Greece	70	45	25	52 ± 12	51 ± 7	37/18	18/7	33.5 ± 7	31 ± 3	1.4 ± 0.9	0.64 ± 0.3	7
Arias	2008	Spain	45	30	15	48 ± 10	52 ± 13	30/0	15/0	30.5 ± 4.0	28.7 ± 4.7	18.5 ± 13.4	11.4 ± 12.2	6
Constantinidis	2008	Greece	51	obses 13; overweighted 11	obses 12; overweighted 15	Mean (range) 45.1 (26–54)	Matched	13/0; 11/0	12/0; 15/0	33.4 ± 1.5; 26.1 ± 1.1	34.9 ± 1.8; 27.4 ± 0.8	124.64 ± 96.7;105 ± 88.7	78.8 ± 50.1;48.5 ± 36.7	5
Kanbay	2008	Turkey	138	106	32	51.39 ± 10.37	44.79 ± 13.35	NR	NR	31.06 ± 5.87	28.85 ± 5.49	114.15 ± 144.15	34.25 ± 13.1	7
Bravo	2007	Spain	70	with EDS 28; without EDS 22	20	51.3 ± 1.4;52.3 ± 2.4	47.4 ± 1.2	28/0; 22/0	20/0	33.3 ± 1.0; 30.9 ± 1.4	28.4 ± 0.6	0.82 ± 0.11;0.89 ± 0.37	0.42 ± 0.11	7
Kobayashi	2006	Japan	51	35	16	51.4 ± 13.1	41 ± 13.1	30/5	13/3	27.9 ± 3.6	27.4 ± 3.7	1.11 ± 0.46	0.62 ± 0.44	5
Ryan	2006	Ireland	96	66 (Mild to Moderate 35, Severe 31)	30	Mild to Moderate 42 ± 8, Severe 43 ± 9	41 ± 8	Mild to Moderate 35/0, Severe 31/0	30/0	Mild to Moderate 32.9 ± 6.03, Severe 32.1 ± 3.5	30.7 ± 3.1	median (interquartile) Mild to Moderate 4.15 (2.71,6.05), Severe 6.19(4.9,7.99)	median (interquartile)3.21 (1.91,3.90)	5
Ciftci	2004	Turkey	65	obese 43	obese 22	49.6 ± 9.1	47.2 ± 10.3	43/0	22/0	31.86 ± 4.11	31.03 ± 3.1	4.6 ± 3.39	3.29 ± 2.13	5
Imagawa	2004	Japan	69	24 Severe	45	NR	NR	NR	NR	28.5 ± 3.6	22.9 ± 2.9	AHI 70–89, 28.6 ± 27.9	25 ± 26.4	4
Minoguchi	2004	Japan	36	24 (Mild 12; Moderate to Severe 12)	12	Mild 51 ± 14.8; Moderate to Severe 49.2 ± 11.7	47.5 ± 11.2	Mild 12/0; Moderate to Severe 12/0	12/0	Mild 26.1 ± 1.3; Moderate to Severe 29.1 ± 2.2	22.3 ± 0.9	Mild 104.2 ± 139.2; Moderate to Severe 501.3 ± 378.8	61.7 ± 40.3	6
Teramoto	2003	Japan	80	40	40	NR	NR	34/6	NR	NR	NR	9500 ± 2200	4400 ± 900	3
Alberti	2003	Italy	38	18	20	52.7 ± 12.0	51.3 ± 13.2	13/5	14/6	26.5 ± 2.2	22.1 ± 3.4	9.7 ± 8.5	6.3 ± 3.0	5
Liu	2000	China	38	22	16	47.4 ± 13.6	47.6 ± 14.7	15/7	11/5	27.58 ± 3.28	23.11 ± 2.96	Plasma 299.09 ± 43.57, PBMC 4165.45 ± 1501.43	Plasma 101.88 ± 21.27, PBMC 1596.25 ± 403.08	4
Vgotzas	1997	USA	22	12	10	40.9 ± 2.2	24.1 ± 0.8	11/1	10/0	40.5 ± 3.2	24.6 ± 0.7	2.51 ± 0.13	1.17 ± 0.1	4

*Abbreviations*: ***NAFLD*** **Non-alcoholic fatty liver disease,** *EDS* Excessive daytime sleepiness, *EBC* **Exhaled breath condensate,** *PBMC* Peripheral blood mononuclear cell, *PHAL* pharyngeal lavage, *GCF* gingival crevicular fluid, *NR* not record, *NOS* Newcastle-Ottawa Scale

*study without control group, data were only used in subgroup meta-analysis

### Meta-analysis and publication bias

**Fig. 2:**
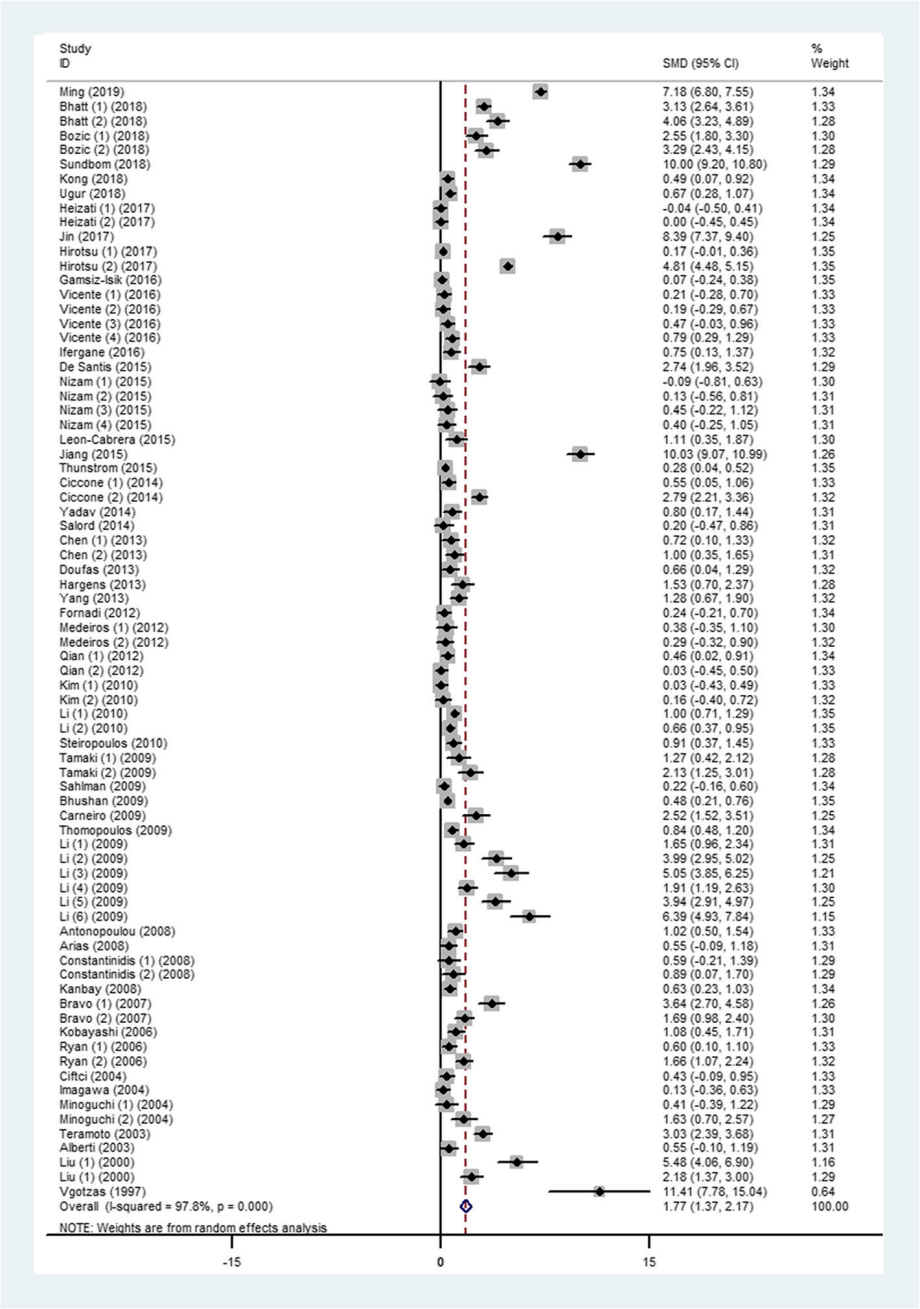
Comparison of TNF-α level between OSA patients and controls in the included studies

**Fig. 3:**
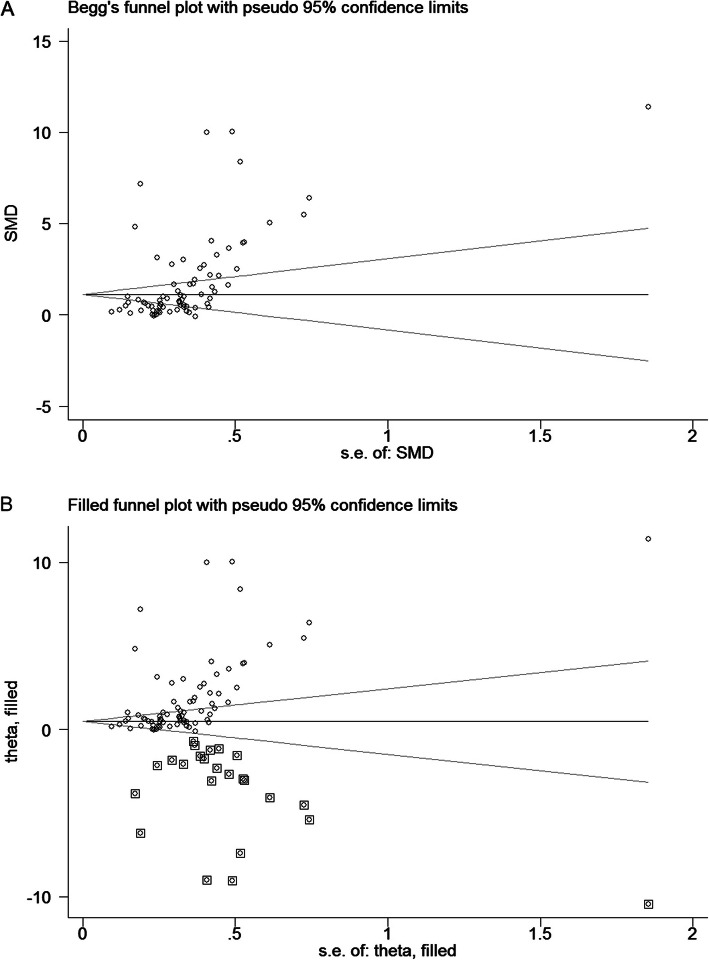
**a**: A funnel plot analysis of publication bias; **b**: A funnel plot adjusted by the trim & fill analysis

The level of TNF-α in OSA patients was 1.77 (95%CI, 1.37 to 2.17, *I*^*2*^ = 97.8%, *P* < 0.0001) times higher than in the control group when all the data from the 50 studies were combined with the random-effects model (Fig. 2). By applying a funnel plot and Egger’s test, publication bias of the literature was evaluated, see Fig. 3a (*t* = 3.33 and *P* = 0.001). Heterogeneity was partially explained by small studies reporting a larger effect and various detection methods and specimens from each study (see subgroup analysis) may have contributed to it. Through omission of each of the included literature studies, results were less changed following sensitivity analysis (Fig. S1, and Table S1). The funnel plots were also adjusted using the trim and fill analysis, see Fig. 3b. By trimming 24 sets of data, the overall SMD (95% CI) changed from 1.768 (1.367 to 2.169) to 1.721 (1.063 to 2.787), which suggesting little evidence of publication bias.

**Fig. 4:**
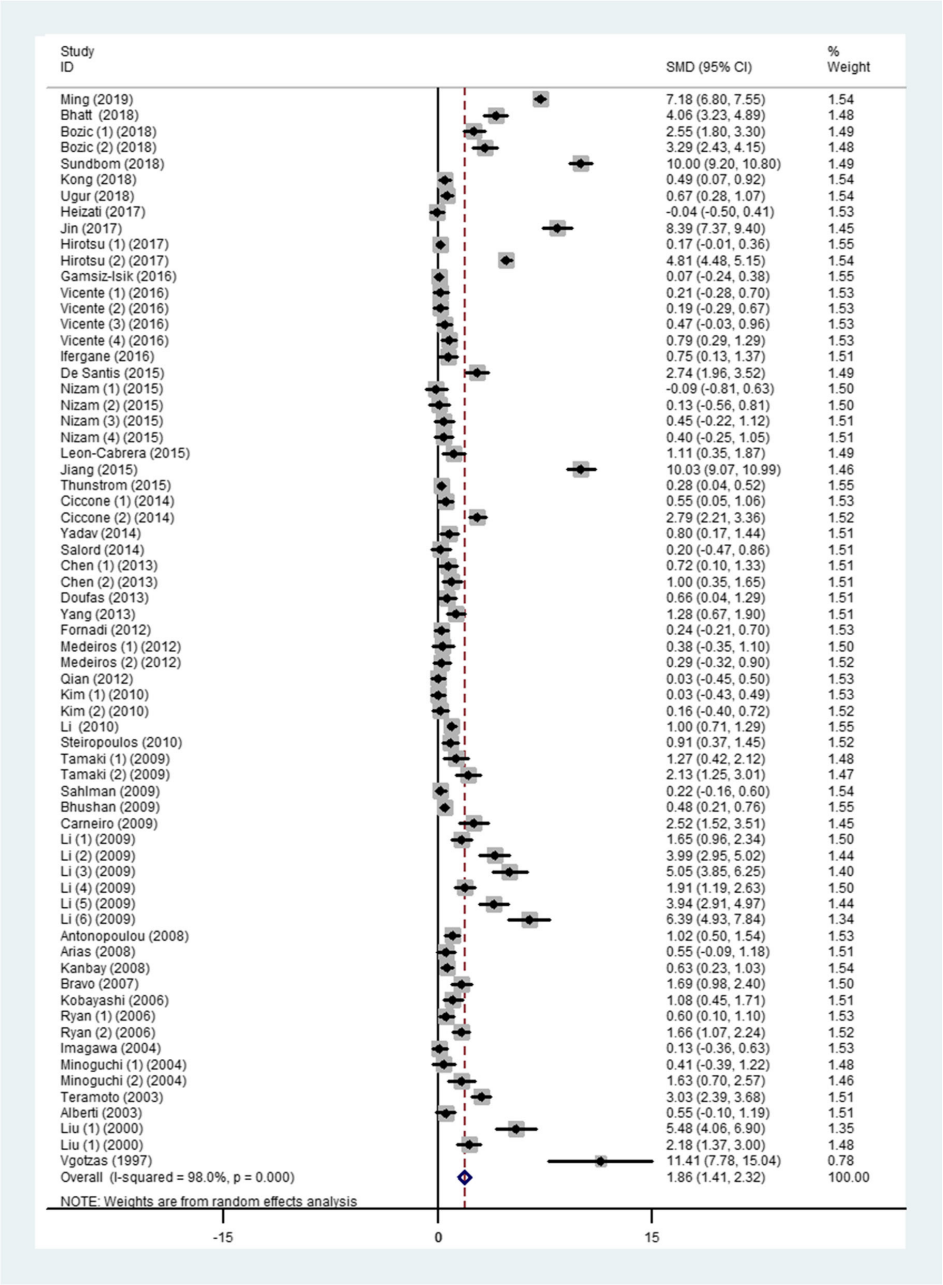
The forest plot for TNF-α between OSA patients and controls by removing articles involved individuals with complications

There were complications mentioned in some of the studies including obese, hypertension, and fatty liver disease. After removal of those articles, the heterogeneity was still significant (SMD 1.86, 95%CI: 1.41 to 2.32, *I*^*2*^ = 98%, *P* < 0.0001), see Fig. 4.

### Subgroup analysis

**Fig. 5:**
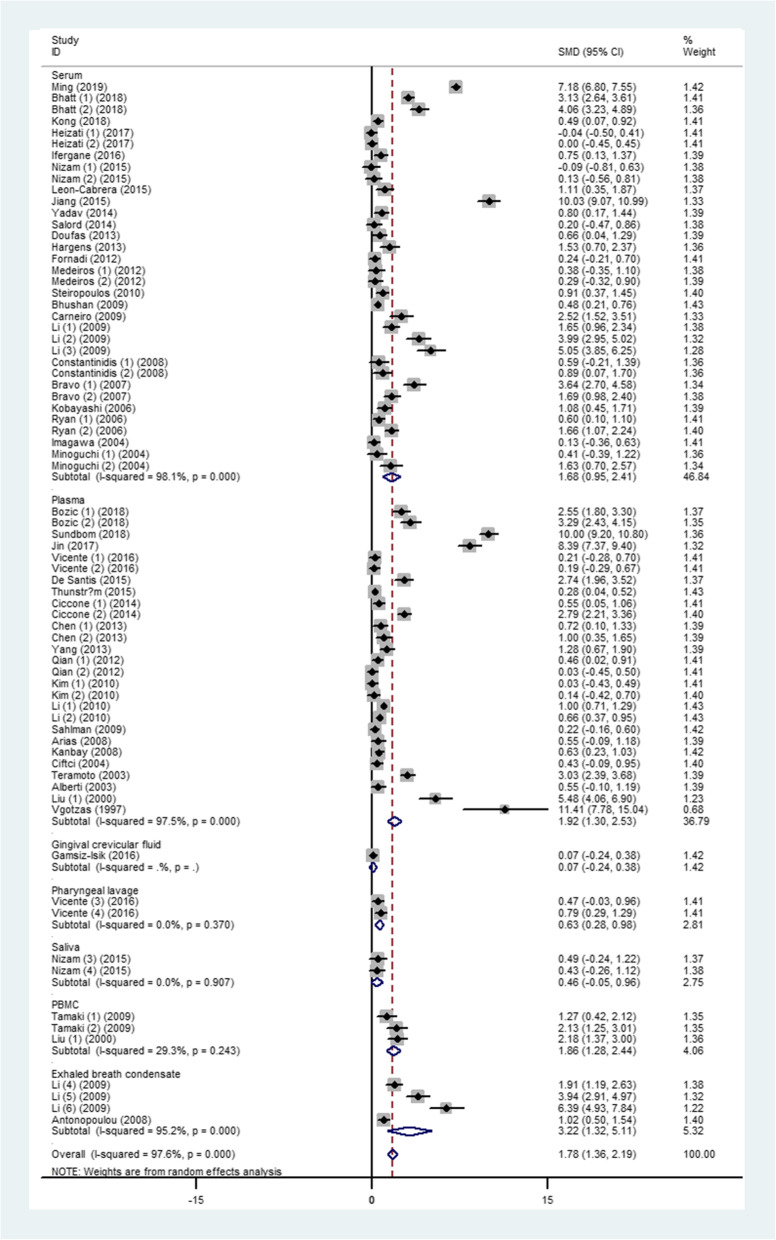
Subgroup analyses of the relationship between TNF-α and OSA according to sample source

Based on the region of each study, the level of TNF-α of OSA patients was 2.56, which was 1.41 times higher than the control group in developing and developed countries, individually (*I*^*2*^ = 98.7%, *P* < 0.0001; *I*^*2*^ = 95.5%, *P* < 0.0001. Fig. S2). The TNF-α was still high in OSA when the individuals were divided into groups from three continents (*P* < 0.0001): Asia: SMD 2.34, 95%CI: 1.67 to 3.01; Europe: SMD 1.44, 95%CI: 0.88 to 2.01; America: SMD 2.15, 95%CI: 0.60 to 3.70 (Fig. S3). Fig. S4 demonstrates that gender did not influence the level of TNF-α in OSA (*P* < 0.0001): males: SMD 1.22, 95%CI: 0.80 to 1.64, and females: SMD 7.39, 95%CI: 2.31 to 12.48. Seven specimens were used for testing the TNF-α level, among which, serum and plasma were commonly used, and the heterogeneity was significant in those two specimen (SMD 1.68, 95%CI: 0.95 to 2.41, *P* < 0.0001; SMD 1.92, 95%CI: 1.30 to 2.53, *P* < 0.0001). However, the TNF-α in the pharyngeal lavage (SMD 0.63, 95%CI: 0.28 to 0.98, *P* = 0.370), saliva (SMD 0.46, 95%CI: − 0.05 to 0.96, *P* = 0.907), and the peripheral blood mononuclear cells (SMD 1.86, 95%CI: 1.28 to 2.44, *P* = 0.243) of OSA had no more statistical significance than they did in counterparts of healthy controls (Fig. 5). Additionally, the TNF-α level in exhaled breath condensate OSA patients were still 3.22 times higher than in control individuals (Fig. 5). For most of the included studies, enzyme linked immunosorbent assay (ELISA) was used to measure the level of TNF-α (SMD 1.85, 95%CI: 1.41 to 2.28, *P* < 0.0001), chemiluminescence analysis (CLIA) was used in three studies (SMD 4.37, 95%CI: 0.58 to 8.16). The remaining two studies were administrated by electrochemiluminescence immunoassay (ECLIA) and polymerase chain reaction (PCR), separately (see Fig. S5).

**Fig. 6:**
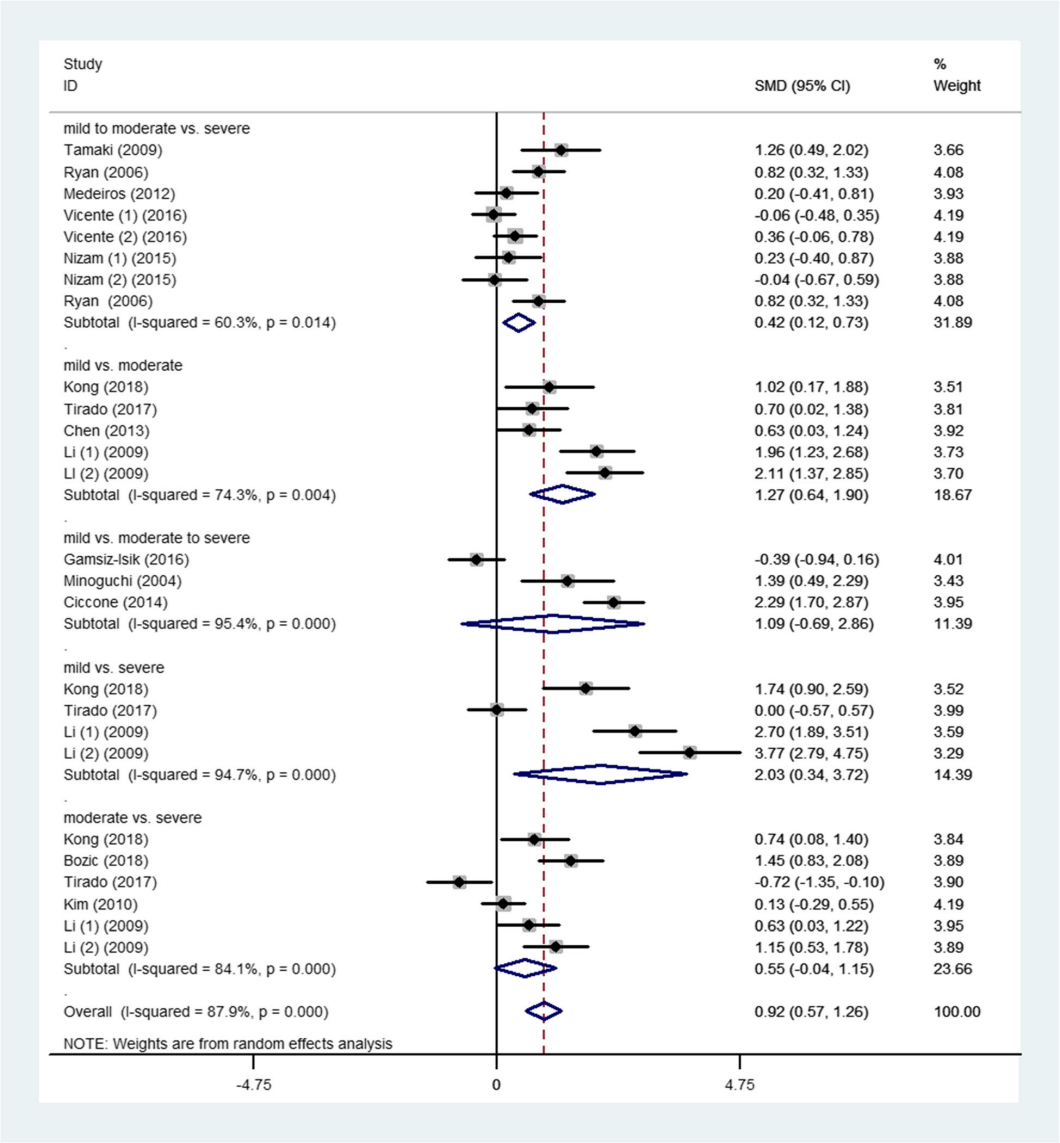
Subgroup analyses of the relationship between TNF-α and OSA according to severity of OSA

Subgroup analysis for the severity of OSA is shown in Fig. 6. Other than the mild to moderate versus the severe group (SMD 0.42, 95%CI: 0.12 to 0.73, *P* = 0.014), other subgroups all reached a statistical significance (Fig. 6).

### Meta-regression

The meta-regression showed that an increase in age of OSA patients significantly predicted an increased effect size (*P* = 0.019). None of the other predictors significantly predicted effect size (Table 2).

**Table 2:** Meta-regression analysis coefficients for TNF-α levels

Covariates	Elements	Coefficient	Standard Error	t	*P*	95% Confidence Interval	
Sample size	–	0.01095	0.00551	1.99000	0.05300	−0.00015	0.02205
BMI	–	0.96000	0.34400	−0.25631	0.72067	0.96000	0.34400
Age	–	0.79053	0.32494	2.43000	0.01900	0.13607	1.44500
NOS	–	−0.57894	0.30767	−1.88000	0.06600	−1.19861	0.04073
Development	–	−1.83779	1.08681	−1.69000	0.09800	−4.02674	0.35115
regions	America	−1.15015	1.41992	−0.81000	0.42200	−4.01001	1.70972
	Asia	−0.18814	1.01291	−0.19000	0.85300	−2.22825	1.85196
	Europe	–	–	–	–	–	–
Severity	All	−0.18343	1.27352	−0.14000	0.88600	−2.74843	2.38157
	mild	−0.45801	1.38464	−0.33000	0.74200	−3.24681	2.33079
	moderate	−0.81363	1.51188	−0.54000	0.59300	−3.85871	2.23145
	moderate to severe	−0.20049	1.32277	−0.15000	0.88000	−2.86469	2.46371
	severe	−0.29741	1.21544	−0.24000	0.80800	−2.74542	2.15061
	mild to moderate	–	–	–	–	–	–
Method	CLIA	0.21982	2.64027	0.08000	0.93400	−5.09796	5.53759
	ELISA	−3.70431	1.97035	−1.88000	0.06700	−7.67281	0.26418
	ECLIA	–	–	–	–	–	–
Specimen	Exhaled breath condensate	2.90545	2.75026	1.06000	0.29600	−2.63385	8.44475
	PBMC	−3.25580	3.41251	−0.95000	0.34500	−10.12894	3.61734
	Pharyngeal lavage	0.79203	2.68878	0.29000	0.77000	−4.62345	6.20751
	Plasma	−0.27113	2.43064	−0.11000	0.91200	−5.16669	4.62444
	Saliva	0.32275	2.90335	0.11000	0.91200	−5.52490	6.17041
	Serum	0.60803	2.45687	0.25000	0.80600	−4.34035	5.55641
	Gingival crevicular fluid	–	–	–	–	–	–
Gender	–	−0.18789	0.35411	−0.53000	0.59800	−0.90109	0.52531
All	–	9.10716	4.37081	2.08000	0.04300	0.30389	17.91043

*Abbreviations*: *NOS* Newcastle-Ottawa Scale, *CLIA* chemiluminescence analysis, *ECLIA* electrochemiluminescence immunoassay, *ELISA* enzyme linked immunosorbent assay, *PBMC* peripheral blood mononuclear cell

## Discussion

Accumulating evidences has proved that airway inflammation had a close connection to airway collapsibility and anatomic narrowing, which were two vital mechanisms involved in the pathogenesis of OSA. Hypopneas and episodes of breathing cessation during sleep caused an increase in pro-inflammatory serum cytokines, conversely, the risk of infection would aggravate collapse of the airway, which causes a decrement in airflow. During the course of this study, we performed a systematic review and meta-analysis to demonstrate the association of elevated TNF-α with OSA. A total of 50 studies were included in the systematic review and meta-analysis. Patients with OSA had significantly (1.77 times) higher TNF-α levels compared with non-OSA patients (95%CI, 1.37 to 2.17, *I*^*2*^ = 97.8%, *P* < 0.0001). On meta-regression, increasing the mean TNF-α level was associated with increased age in OSA patients (Coefficient [Q]: 0.79, 95% CI, 0.14–1.45, *P* = 0.019), and the TNF-α level was consistently correlated with the severity of OSA in the subgroup analysis. TNF-α should be incorporated into a sleep apnea scoring system to stratify the patients for early recognition of severe OSA.

TNF-α possessed a wide range of pro-inflammatory activities and the inappropriate production of TNF was the origin to several diseases, including OSA. In 1997, Vgontzas and colleagues were the first to point that elevated TNF-α was linked to disorders of excessive daytime sleepiness (EDS) [61, 65]. It illustrated that TNF-α was involved in the regulation of physiological sleep, and increased secretion might be associated with sleepiness and fatigue [65]. Subsequent studies demonstrated that an elevated TNF-α level in OSA was independently of EDS, also, a marker of cardiovascular pathophysiology in OSA [50]. Furthermore, continuous positive airway pressure (CPAP) therapy could suppress the of secretion TNF-α, and when OSA patients received anti-TNF-α therapy, significant reduction occurred in daytime sleepiness and AHI [5, 66].

Previous meta-analysis studies have evaluated the association between TNF-α levels and OSA, however, they only involved pediatric OSA individuals and less studies were collected [5, 67]. Infants and juveniles were still in a stage of growth and development, the immune systems and inflammation environments of airway were different from adults. The clinical manifestations, predisposing factors, and polysomnographic data in children with OSA were also different from those in adults. If it was calculated with all-age adults, it definitely affected the credibility and accuracy of the statistical results. Additionally, the units of TNF-α levels were not uniformed, and detected methods and specimen were not analyzed, which may cause bring heterogeneity. Consider all of the above, we updated this meta-analysis to remedy the limitations and summarized the connection between the two.

In conjunction with previous research, the current meta-analysis demonstrates that the TNF-α levels were higher in patients with OSA than in healthy controls. By excluding complications such as obesity (over weight), hypertension, and fatty liver disease, the levels were still 1.852 times higher (*P* < 0.0001). Because a substantial heterogeneity existed in the test results, we further performed the subgroup analysis. The results showed the magnitude of the effect was consistent across the subgroups examined, as the coefficients for gender, region and development of countries were significant. As for the female OSA patients, although only 2 studies were added to this analysis, the level of TNF-α was more pronounced than in the male group when compared with healthy individuals [34, 54]. Women had long been generally underrepresented for OSA, with most sleep research focusing on OSA in males [68]. This gender difference reflected in the menstrual cycle, pregnancy, and menopause, resulting in secretion and fluctuation of sex hormones, therefore, this gender-related regulation participated in mechanisms of sleep disorders [69]. Vgontzas et al. reported that TNF-α was elevated in women with polycystic ovary syndrome, but it was not associated with OSA, unlike in male [70]. Further insightful studies should be devoted to the pathophysiology of females with OSA to identify the mechanism of this gender difference.

Obesity is regarded as a chronic low-grade inflammatory state. Obesity had been shown to have an effect on pulmonary functions, affecting the residual volume and total lung capacity, and was considered to be a risk factor of OSA [71]. Considering the possible mechanism, TNF-α is one of the key pro-inflammatory cytokines. In the inflammatory condition, hypoxia of adipocytes is related to macrophage infiltration, which is the most abundant type of immune cell in adipose tissue. Together with macrophages, adipocytes release various biologically active molecules to affect the innate and adaptive immune system, such as cytokines, chemokines, complement proteins, and other acute-phase proteins (also known as adipokines). These secretions further contributed to maintain the activation of immune cells and their infiltration into regulatory organs [72]. Based on a number of laboratory data and clinical evidence, TNF-α inhibitors were implied as new therapeutic strategies in several immune-related diseases, including rheumatoid arthritis, asthma, sarcoidosis, inflammatory bowel disease, pancreatitis, and so on [73–75].

Studies had investigated the effects of weight loss on measures of airway inflammation [76]. In this meta-analysis, we analyzed related confounding factors through meta regression, however, BMI did not reach statistical significance. Meanwhile, obesity is also associated with increased numbers of adipose tissue macrophages. Especially in the visceral adipose tissue depot, an increasing number of adipose tissue macrophages were showing even more in the presence of abdominal obesity. And, along with weight reduction, a decrease in adipose tissue macrophages numbers can be observed [77]. The prevalence of obesity and inflammatory also raises steadily among older age groups, especially a fat deposition in abdominal obesity, a major contributor to the metabolic syndrome and age-related disease, rather than subcutaneous fat. Moreover, aging is associated with higher levels of pro-inflammatory cytokines. The fat mass redistribution and increased fat mass accumulation with age lead to disregulation of cytokines and adipokine secretion which linked to key features of local and systemic inflammation, such as TNF-α. Increasing the incidence of obesity among the elderly may probably intensify and accelerate the problem of age-related inflammation processes [78]. Considering the close relationships among age, obesity, and immune balances, we preferred to believe the immune intervening method, taking TNF-α inhibitors as the typical example, would provide new insight into the treatment of OSA.

Past studies had demonstrated that proinflammatory cytokines were consistently correlated with severity of OSA [42, 44]. This phenomenon was also shown in TNF-α in the current study. The specimen source used for measurement of TNF-α was seldom discussed, and most of the previous studies used peripheral blood. Exhaled breath condensate (EBC) is a non-invasive and safe method to monitor inflammation, and it has already been used to measure biomarkers of inflammation in several respiratory diseases, such as asthma and chronic obstructive disease [44]. Some research has validated that it could be a biomarkers to predict OSA. Using a subgroup analysis, TNF-α levels in EBC had equal effectiveness as in blood specimens, which may suggest its value in predicting severity of OSA by EBC.

Because of the heterogeneity in this meta-analysis, we performed a meta-regression, and the results showed age was significant associated with the effects estimate. Because all the OSA patients were adults, there was still a large span in age in our meta-analysis. The prevalence of OSA tended to increase with age, whereas, the clinical severity was reversed in available studies [79–81]. The speculative explanations may be attributed to influence of BMI on the severity of OSA, and the high collapsibility of the upper airways in the elderly. Additionally, the inclusion criteria varied among each other; hence some unavoidable complications could have been introduced into the study samples.

In this study, we tried to eliminate utmost confounding factors to draw a reliable conclusion. However, there were still several limitations that should be addressed. First, because of the number of studies that we analyzed, publishing bias inevitably existed. Even though we had contacted with the authors for additional details regarding the studies, data were still unavailable from the literatures. Second, the inflammation fluctuated in 1 d; additionally, the times of drawing blood were not stable, which meant TNF-α levels were not measured on the same baseline, for which a difference occurred throughout the study population. Third, diet, lifestyles, environmental factors, etc., all related to the state of OSA, in turn, affecting the secretion of inflammatory factors in the body.

## Conclusion

In conclusion, this meta-analysis identified a significant association between OSA and elevated TNF-α level in adults and was consistently correlated with severity of OSA. Although the above findings need to be confirmed in larger case-control cohorts, they guides the way to seeking a potential biomarker and therapeutic target in adults with OSA. Further studies are needed to better standardized detection of inflammatory cytokines (TNF-α) and establish an exact link to the assessment of OSA.

## Supplementary information

**Additional file 1: Table S1.** Sensitivity analysis by omitting each of the included literatures.

**Additional file 2: Figure S1.** Sensitivity analysis.

**Additional file 3: Figure S2.** Subgroup analyses of the relationship between TNF-α and OSA according to development of country.

**Additional file 4: Figure S3.** Subgroup analyses of the relationship between TNF-α and OSA according to continent.

**Additional file 5: Figure S4.** Subgroup analyses of the relationship between TNF-α and OSA according to gender.

**Additional file 6: Figure S5.** Subgroup analyses of the relationship between TNF-α and OSA according to laboratory examination.

## Data Availability

All data and material in this paper could achieve in supplemental materials.

## Abbreviations

AHI: Apnea-hypopnea index
BMI: Body mass index
CPAP: Continuous positive airway pressure
CLIA: Chemiluminescence analysis
EBC: Exhaled breath condensate
EDS: Excessive daytime sleepiness
ECLIA: Electrochemiluminescence immunoassay
ELISA: Enzyme linked immunosorbent assay
GCF: Gingival crevicular fluid
NAFLD: Non-alcoholic fatty liver disease
NOS: Newcastle-Ottawa Scale
NR: Not record
OSA: Obstructive sleep apnea
PBMC: Peripheral blood mononuclear cell
PHAL: Pharyngeal lavage
PICOS: Population, intervention, comparison, outcomes, and study design
SMD: Standard mean difference
TNF-α: Tumor necrosis factor-α
