# Supplementary material for: Association between tumor necrosis factor alpha and obstructive sleep apnea in adults: a meta-analysis update
Source: BMC Pulm Med. 2020 Aug 12;20:215. doi: 10.1186/s12890-020-01253-0 (PMC7425010; doi:10.1186/s12890-020-01253-0)
Supplement: Supplementary file 1 — Additional file 1: Table S1. Sensitivity analysis by omitting each of the included literatures. [file 12890_2020_1253_MOESM1_ESM.doc]

| Table S1. Sensitivity analysis by omitting each of the included literatures. |
| --- |

| Study omitted | Estimate | 95% Conf. Interval | |
| --- | --- | --- | --- |
| Ming (2019) | 1.6702348 | 1.326143 | 2.0143266 |
| Bhatt (1) (2018) | 1.7493769 | 1.3469633 | 2.1517906 |
| Bhatt (2) (2018) | 1.737581 | 1.3361124 | 2.1390495 |
| Bozic (1) (2018) | 1.7578752 | 1.3542166 | 2.1615338 |
| Bozic (2) (2018) | 1.7481276 | 1.345384 | 2.150871 |
| Sundbom (2018) | 1.650908 | 1.2746508 | 2.0271652 |
| Kong (2018) | 1.7864273 | 1.3797803 | 2.193074 |
| Ugur (2018) | 1.7842783 | 1.3768444 | 2.1917121 |
| Heizati (1) (2017) | 1.7931347 | 1.387993 | 2.1982763 |
| Heizati (2) (2017) | 1.7926353 | 1.387308 | 2.1979628 |
| Jin (2017) | 1.6807494 | 1.2882184 | 2.0732803 |
| Hirotsu (1) (2017) | 1.7940357 | 1.3780274 | 2.2100441 |
| Hirotsu (2) (2017) | 1.7183974 | 1.337293 | 2.0995018 |
| Gamsiz-Isik (2016) | 1.7927104 | 1.3848746 | 2.2005463 |
| Vicente (1) (2016) | 1.7897066 | 1.3843238 | 2.1950891 |
| Vicente (2) (2016) | 1.7900831 | 1.3846536 | 2.1955125 |
| Vicente (3) (2016) | 1.7863469 | 1.3806485 | 2.1920455 |
| Vicente (4) (2016) | 1.7821079 | 1.3761342 | 2.1880817 |
| Ifergane (2016) | 1.7821139 | 1.3771375 | 2.1870904 |
| De Santis (2015) | 1.7553988 | 1.3519781 | 2.1588194 |
| Nizam (1) (2015) | 1.792729 | 1.3887838 | 2.1966741 |
| Nizam (2) (2015) | 1.7900836 | 1.3858337 | 2.1943336 |
| Nizam (3) (2015) | 1.7859446 | 1.381377 | 2.1905122 |
| Nizam (4) (2015) | 1.7865974 | 1.3819798 | 2.191215 |
| Leon-Cabrera (2015) | 1.7770588 | 1.3726463 | 2.1814713 |
| Jiang (2015) | 1.6563534 | 1.271588 | 2.0411186 |
| Thunstrom (2015) | 1.7912542 | 1.3790921 | 2.2034163 |
| Ciccone (1) (2014) | 1.7851861 | 1.3794675 | 2.1909046 |
| Ciccone (2) (2014) | 1.754467 | 1.3509496 | 2.1579843 |
| Yadav (2014) | 1.7813905 | 1.3764949 | 2.1862862 |
| Salord (2014) | 1.7892436 | 1.3848656 | 2.1936216 |
| Chen (1) (2013) | 1.7826297 | 1.3776653 | 2.1875942 |
| Chen (2) (2013) | 1.778748 | 1.3738785 | 2.1836176 |
| Doufas (2013) | 1.7833226 | 1.3784192 | 2.188226 |
| Hargens (2013) | 1.7713797 | 1.3672372 | 2.1755221 |
| Yang (2013) | 1.7750899 | 1.3699979 | 2.180182 |
| Fornadi (2012) | 1.7894959 | 1.3837067 | 2.1952851 |
| Medeiros (1) (2012) | 1.7867426 | 1.3824184 | 2.1910667 |
| Medeiros (2) (2012) | 1.7882723 | 1.3835561 | 2.1929884 |
| Qian (1) (2012) | 1.7866637 | 1.3803827 | 2.1929448 |
| Qian (2) (2012) | 1.7921865 | 1.3870201 | 2.1973529 |
| Kim (1) (2010) | 1.7921872 | 1.386919 | 2.1974554 |
| Kim (2) (2010) | 1.7901186 | 1.3852962 | 2.194941 |
| Li (1) (2010) | 1.78118 | 1.3697199 | 2.1926403 |
| Li (2) (2010) | 1.7855591 | 1.3748652 | 2.1962531 |
| Steiropoulos (2010) | 1.7803218 | 1.3747482 | 2.1858954 |
| Tamaki (1) (2009) | 1.7748036 | 1.3706542 | 2.1789532 |
| Tamaki (2) (2009) | 1.7635452 | 1.3597459 | 2.1673443 |
| Sahlman (2009) | 1.7902721 | 1.3834591 | 2.1970851 |
| Bhushan (2009) | 1.7880975 | 1.3771495 | 2.1990454 |
| Carneiro (2009) | 1.7586048 | 1.3551855 | 2.162024 |
| Thomopoulos (2009) | 1.7824082 | 1.3738815 | 2.1909349 |
| Li (1) (2009) | 1.7699995 | 1.36543 | 2.1745691 |
| Li (2) (2009) | 1.7397009 | 1.337585 | 2.1418169 |
| Li (3) (2009) | 1.7272239 | 1.3259976 | 2.1284502 |
| Li (4) (2009) | 1.7664686 | 1.3621608 | 2.1707764 |
| Li (5) (2009) | 1.7402214 | 1.3380674 | 2.1423755 |
| Li (6) (2009) | 1.7133725 | 1.3129454 | 2.1137996 |
| Antonopoulou (2008) | 1.7789497 | 1.3731167 | 2.1847827 |
| Arias (2008) | 1.7848251 | 1.3800164 | 2.1896338 |
| Constantinidis (1) (2008) | 1.7837514 | 1.379565 | 2.1879377 |
| Constantinidis (2) (2008) | 1.7798482 | 1.3756261 | 2.1840703 |
| Kanbay (2008) | 1.7847686 | 1.3775764 | 2.1919608 |
| Bravo (1) (2007) | 1.7438333 | 1.3414413 | 2.1462255 |
| Bravo (2) (2007) | 1.769502 | 1.3650314 | 2.1739728 |
| Kobayashi (2006) | 1.7777759 | 1.3727766 | 2.1827753 |
| Ryan (1) (2006) | 1.7846127 | 1.3788089 | 2.1904166 |
| Ryan (2) (2006) | 1.7701116 | 1.3650107 | 2.1752124 |
| Ciftci (2004) | 1.7867178 | 1.3812759 | 2.1921597 |
| Imagawa (2004) | 1.7907019 | 1.3854997 | 2.195904 |
| Minoguchi (1) (2004) | 1.7859672 | 1.3818702 | 2.1900642 |
| Minoguchi (2) (2004) | 1.7700076 | 1.3661129 | 2.173902 |
| Teramoto (2003) | 1.7510884 | 1.3480977 | 2.1540792 |
| Alberti (2003) | 1.7847627 | 1.3800458 | 2.1894796 |
| Liu (1) (2000) | 1.7239165 | 1.3226529 | 2.12518 |
| Liu (1) (2000) | 1.7628113 | 1.3589398 | 2.166683 |
| Vgotzas (1997) | 1.7054702 | 1.3052386 | 2.1057019 |
| Combined | 1.7680041 | 1.36744 | 2.1685681 |
