# Supplementary figures and images for: Association between tumor necrosis factor alpha and obstructive sleep apnea in adults: a meta-analysis update
Source: BMC Pulm Med. 2020 Aug 12;20:215. doi: 10.1186/s12890-020-01253-0 (PMC7425010; doi:10.1186/s12890-020-01253-0)

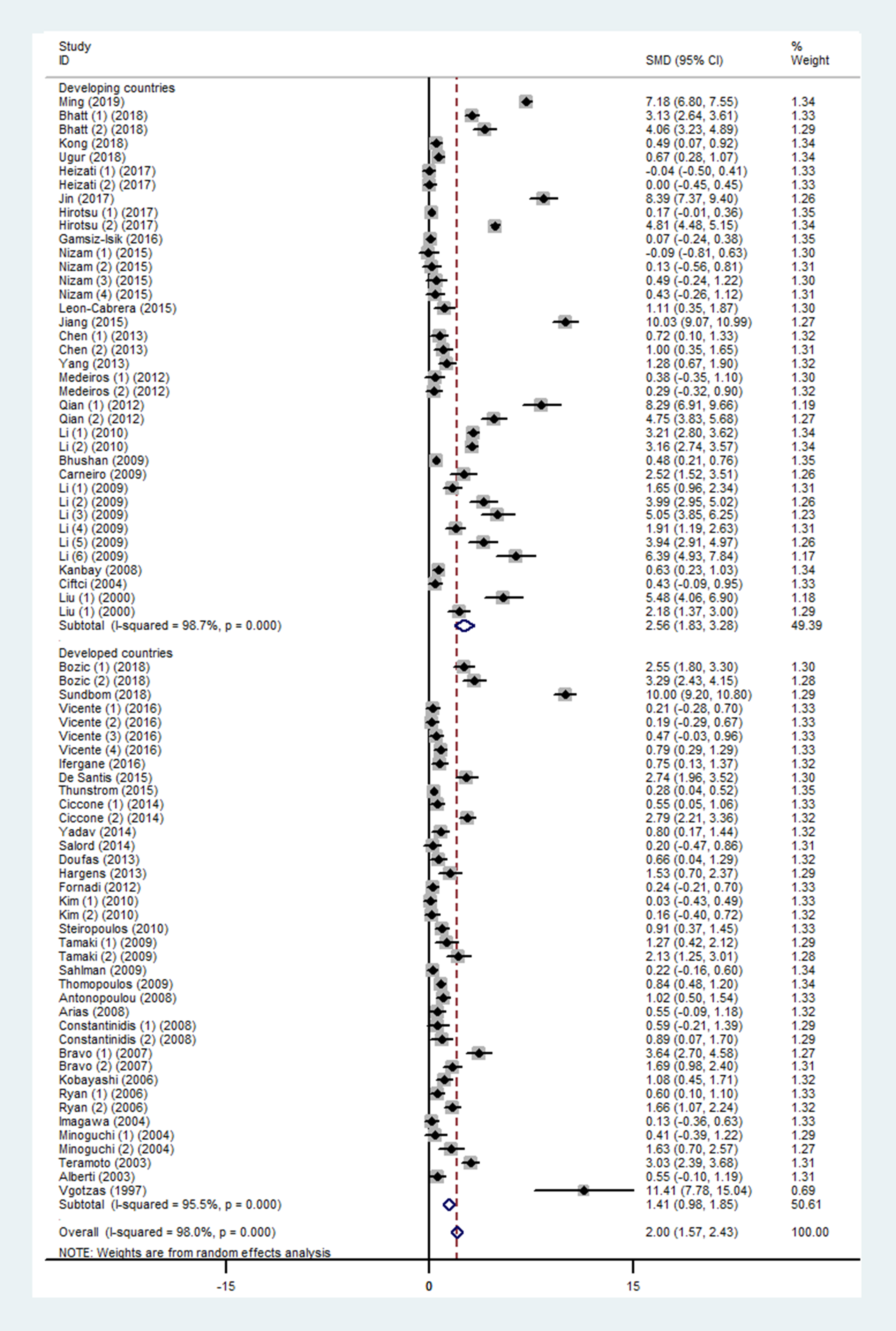

Supplement: Supplementary file 3 — Additional file 3: Figure S2. Subgroup analyses of the relationship between TNF-α and OSA according to development of country. [file 12890_2020_1253_MOESM3_ESM.jpg]

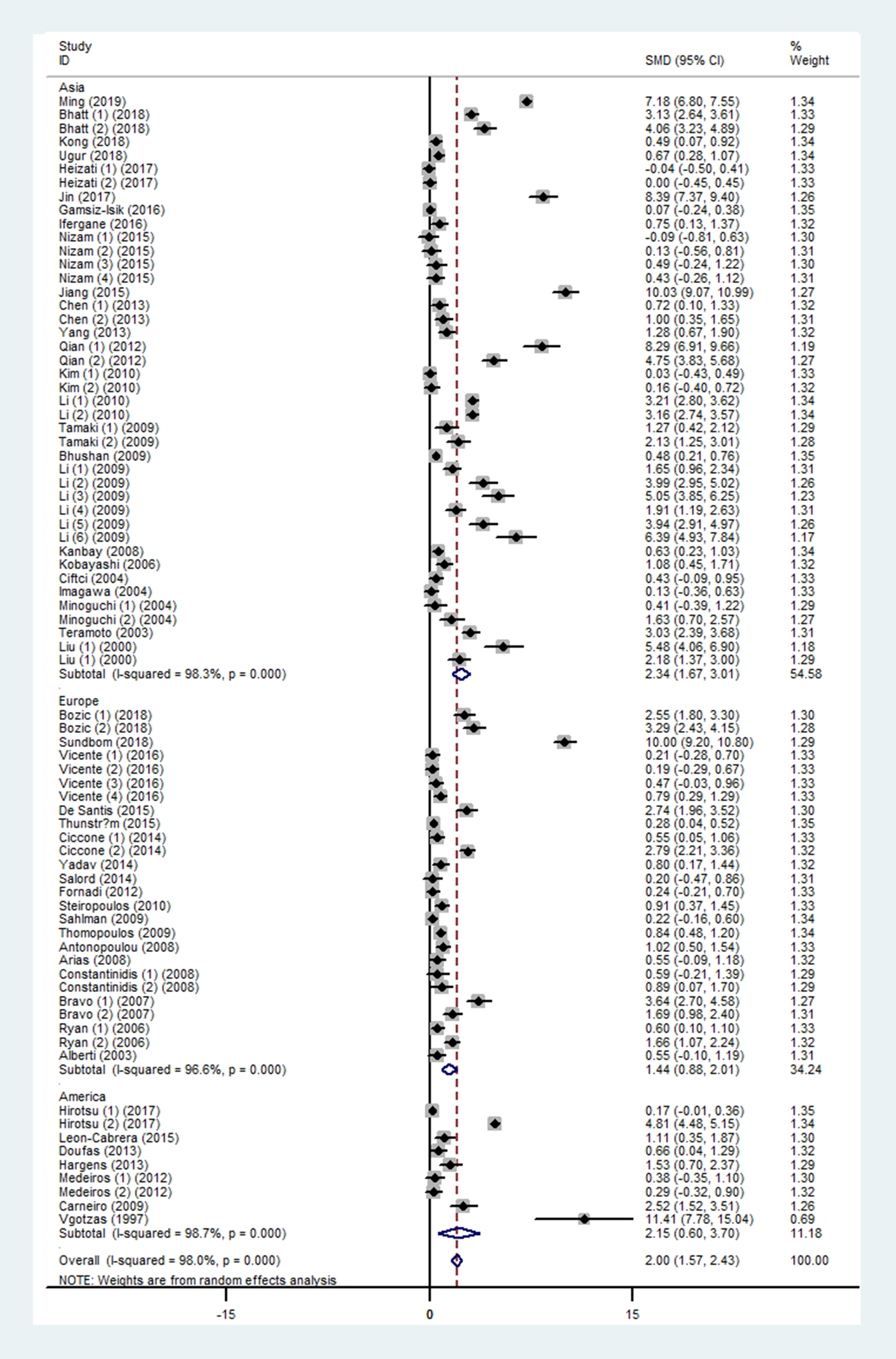

Supplement: Supplementary file 4 — Additional file 4: Figure S3. Subgroup analyses of the relationship between TNF-α and OSA according to continent. [file 12890_2020_1253_MOESM4_ESM.jpg]

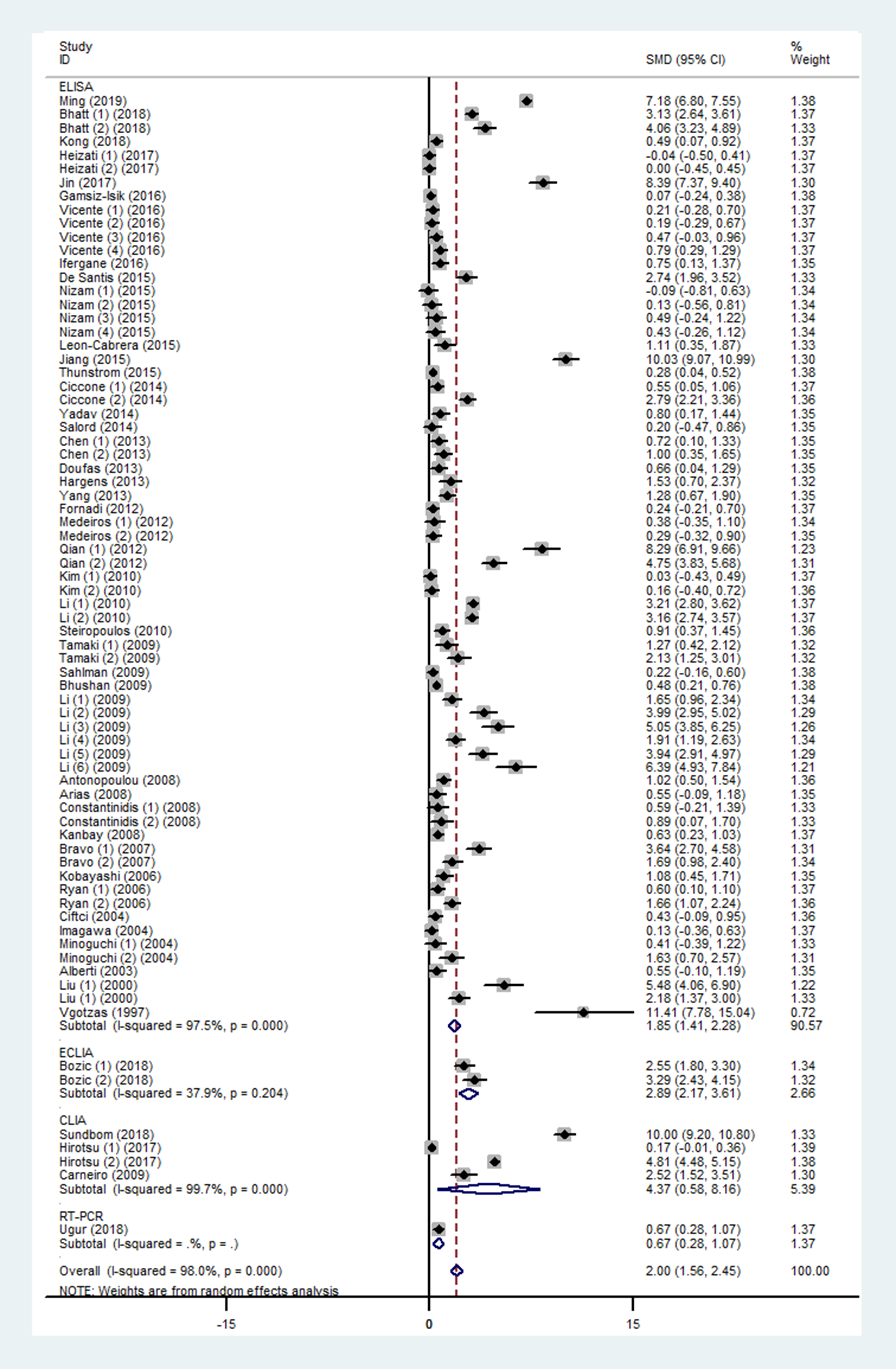

Supplement: Supplementary file 6 — Additional file 6: Figure S5. Subgroup analyses of the relationship between TNF-α and OSA according to laboratory examination. [file 12890_2020_1253_MOESM6_ESM.jpg]
